# Supplementary material for: Past and present giant viruses diversity explored through permafrost metagenomics
Source: Nat Commun. 2022 Oct 7;13:5853. doi: 10.1038/s41467-022-33633-x (PMC9546926; doi:10.1038/s41467-022-33633-x)
Supplement: Supplementary file 1 — Supplementary Information [file 41467_2022_33633_MOESM1_ESM.pdf]

Supplementary Information file

Past and present giant viruses diversity explored through permafrost metagenomics

Sofia Rigou[1], Sébastien Santini[1], Chantal Abergel[1], Jean-Michel Claverie[1], Matthieu Legendre[1,*]

[1]Aix–Marseille University, Centre National de la Recherche Scientifique, Information Génomique & Structurale, Unité Mixte de Recherche 7256 (Institut de Microbiologie de la Méditerranée, FR3479), 13288 Marseille Cedex 9, France

[*]Correspondence: legendre@igs.cnrs-mrs.fr

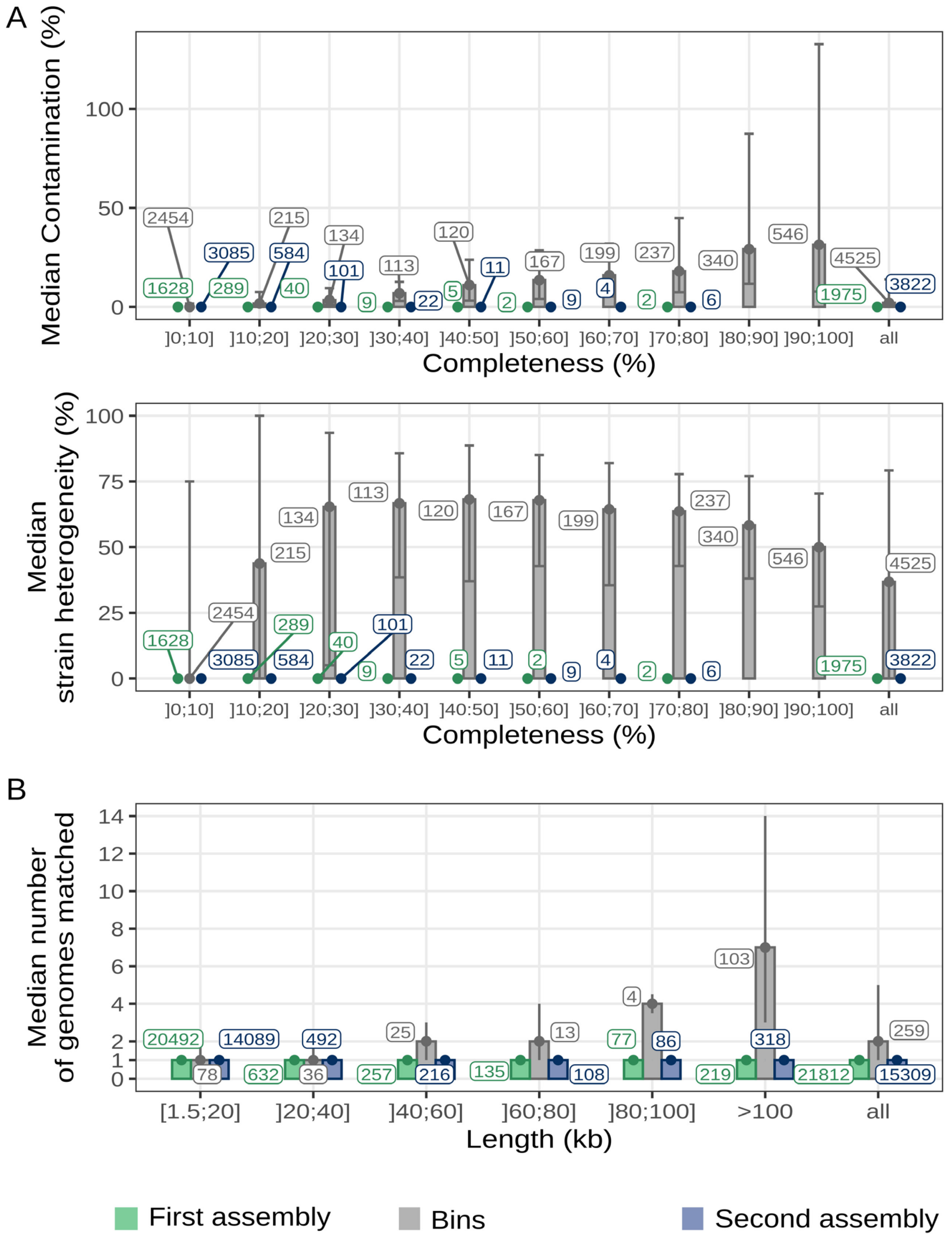

Supplementary Figure 1. Control for chimerism
CheckM was ran on the (A) 11 Russian cryosol samples to check for chimeras in contigs (first assembly), bins or scaffolds (second assembly). (B) Three MOCK complex communities were assembled using the same methodology. The resulting sequences were then aligned to their references by BLASTN to assess their level of chimerism (i.e. matching several genomes). Points indicate median values and the error bars correspond to the 25% and 75% quantiles. In the case of n < 3, no median was computed as all points correspond to zero contamination and zero strain heterogeneity. Counts are shown on top of each bar. Source data are provided as a Source Data file.

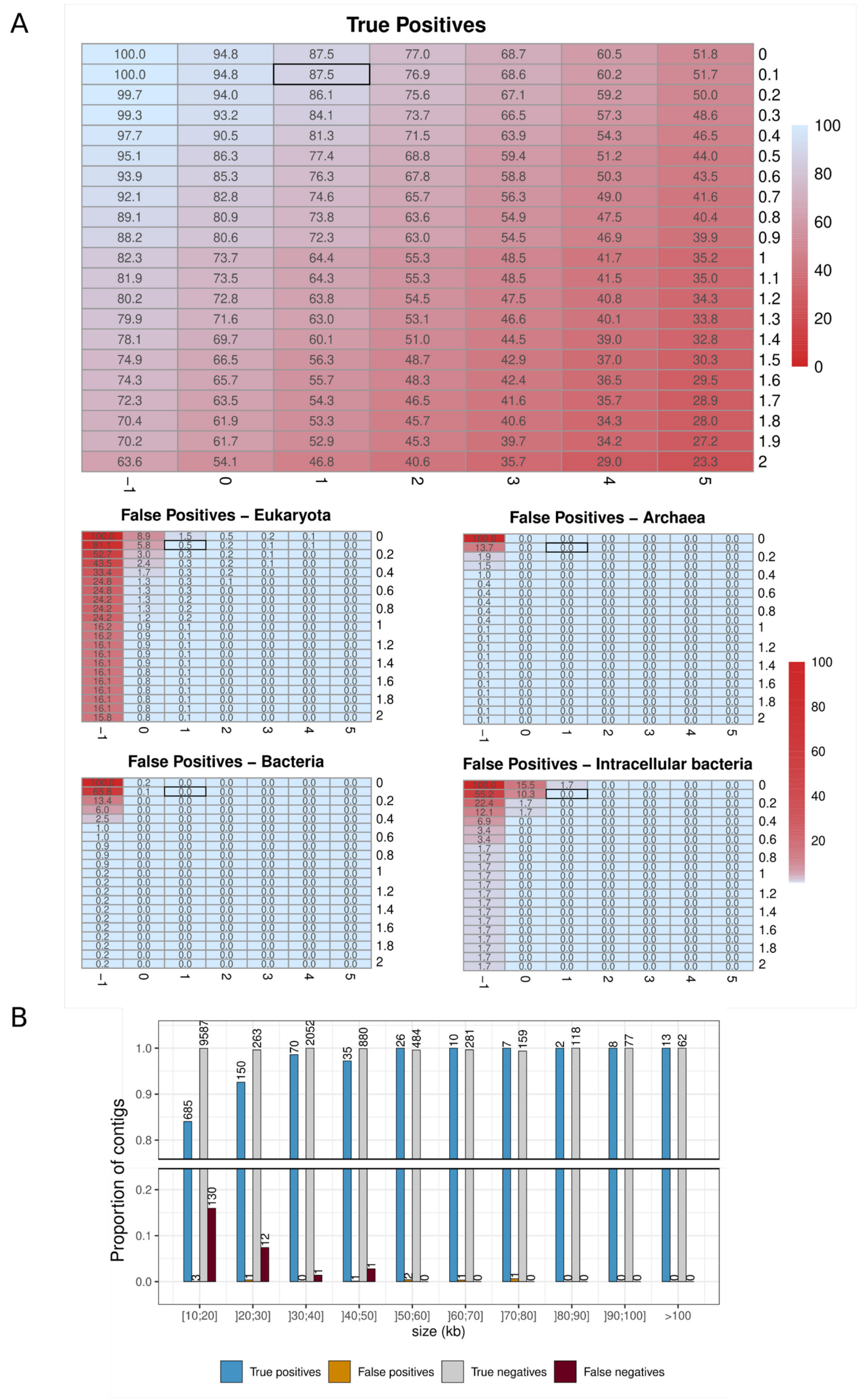

Supplementary Figure 2. Tested threshold parameters for *Nucleocytoviricota* sequence retrieval on a control dataset

(A) From a control dataset (see methods) parameters were estimated to discriminate viral and cellular contigs. The intercept parameter is shown on the x-axis and the slope on the y-axis. Notice that only eukaryotes render false positives at the optimal parameters (x=1, y=0.1). (B) Applying the threshold resulted in false/true positives and false/true negatives that were then computed for different contig sizes. The number of contigs is written above each bar.

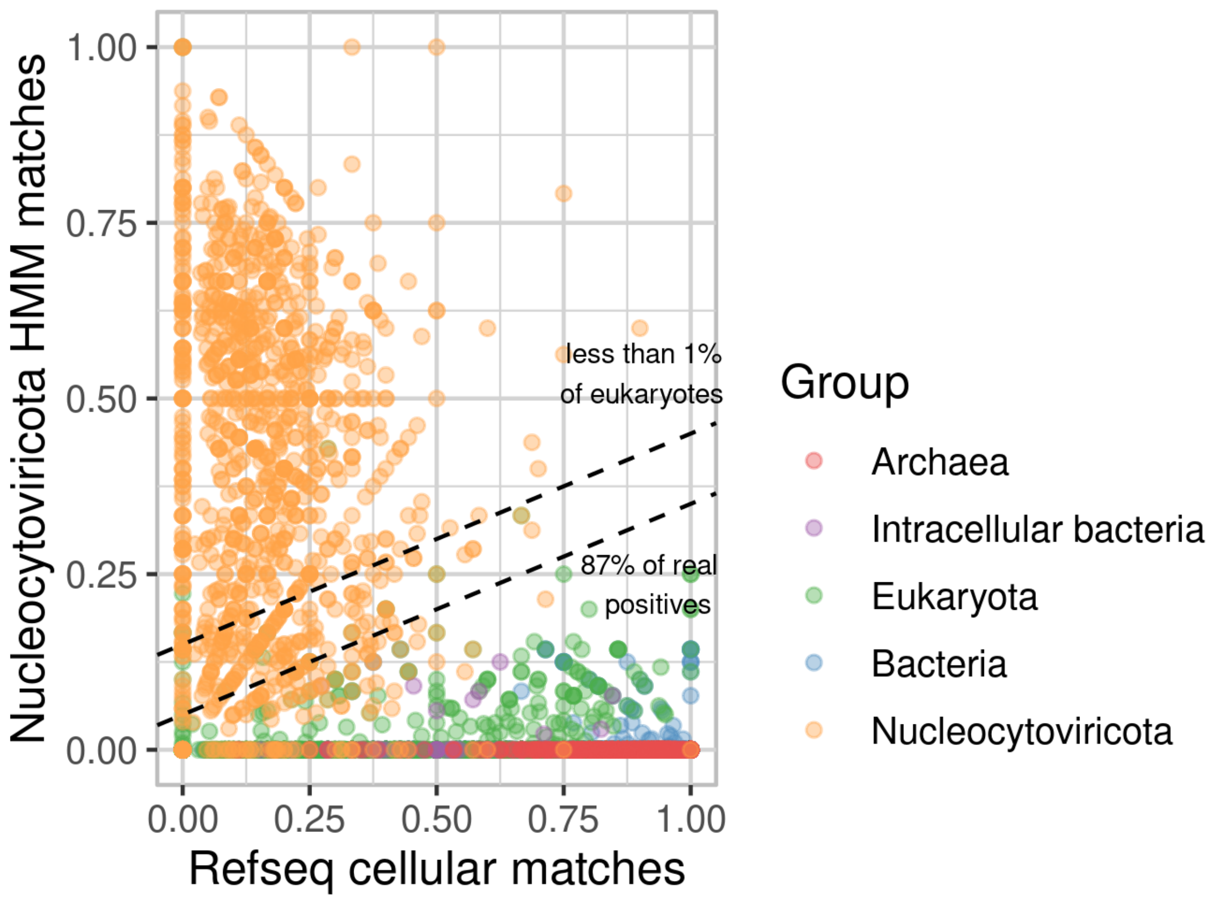

Supplementary Figure 3. Filtering of scaffolds according to their proportion of viral and cellular matches

An alternative methodology was tested in which we used the proportions instead of the number of matches. All tested parameters for determining the threshold resulted to be suboptimal compared to the previous methodology (Fig. 1 in the main text) with either more false positives (the lowest dashed line) or more false negatives (the upper dashed line). Source data are provided as a Source Data file.

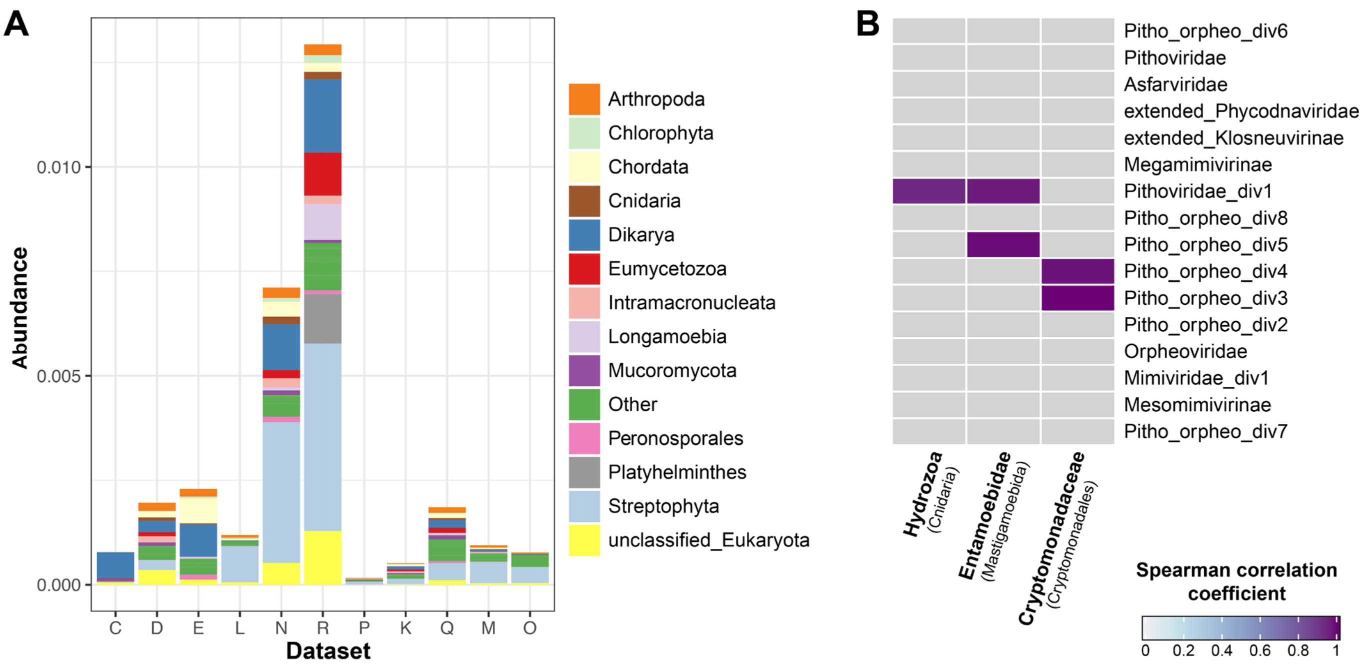

Supplementary Figure 4. Eukaryotic abundance across samples and co-occurrence with viruses

(A) The relative abundance of a given eukaryotic group is given as the sum of coverages of the sequences belonging to it divided by the total sample coverage. (B) Co-occurrence between viral and eukaryotic families. The spearman correlations were calculated from the estimated normalized abundance (sum of coverages) in each sample. Two-sided correlation test p-values were corrected for multiple testing using the Benjamini & Hochberg FDR correction. Only significantly co-occurring (corrected p-value < 0.01) Eucaryote/virus pairs are color coded. Source data are provided as a Source Data file.

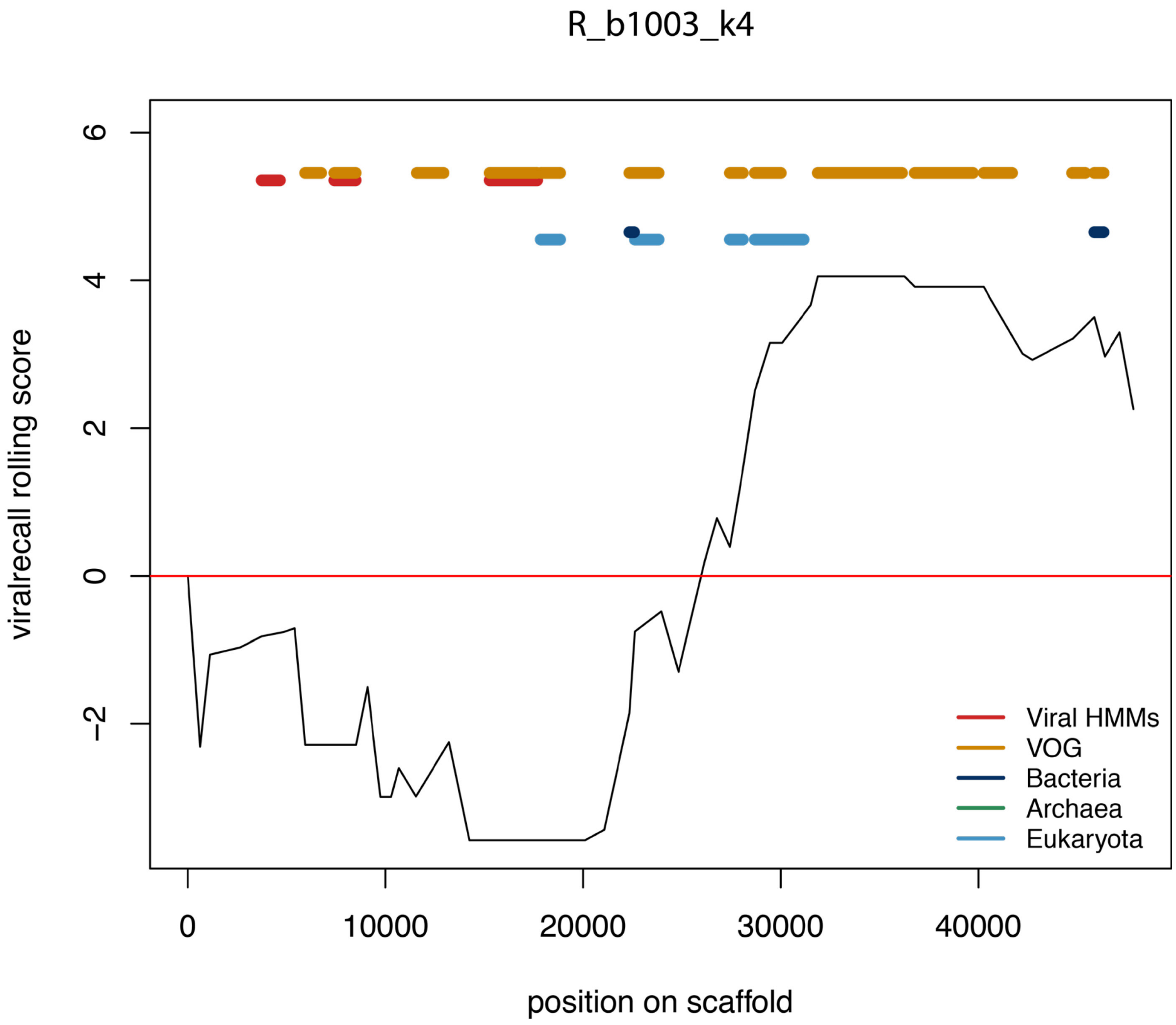

Supplementary Figure 5. Identification of potentially endogenized viruses using ViralRecall

The red line indicates the decision threshold between potentially cellular (negative) or viral (positive) genomic regions as determined by ViralRecall. In this example, the putative cellular portion does have matches in the VOG database and its DIAMOND BLASTP cellular matches are both from Eukaryotes and Bacteria.

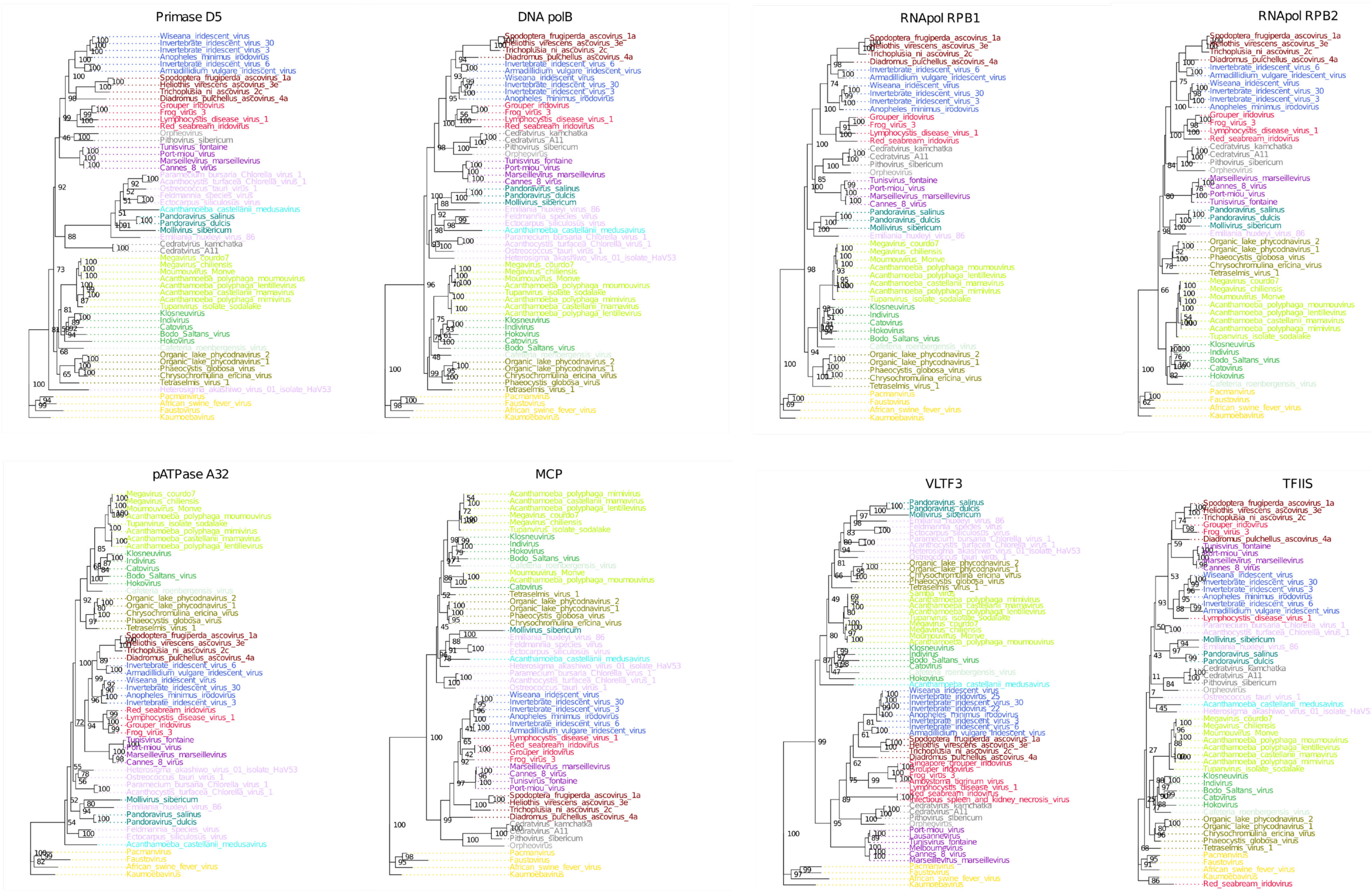

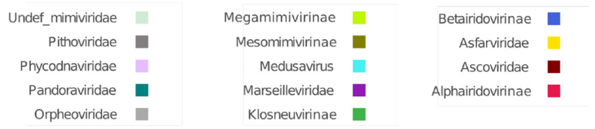

Supplementary Figure 6. Phylogenetic trees of marker genes from the reference *Nucleocytoviricota* genomes

Consensus trees (parameters –bb 1000 –bi 100) and optimal models (-m MFP) were calculated by IQ-TREE.

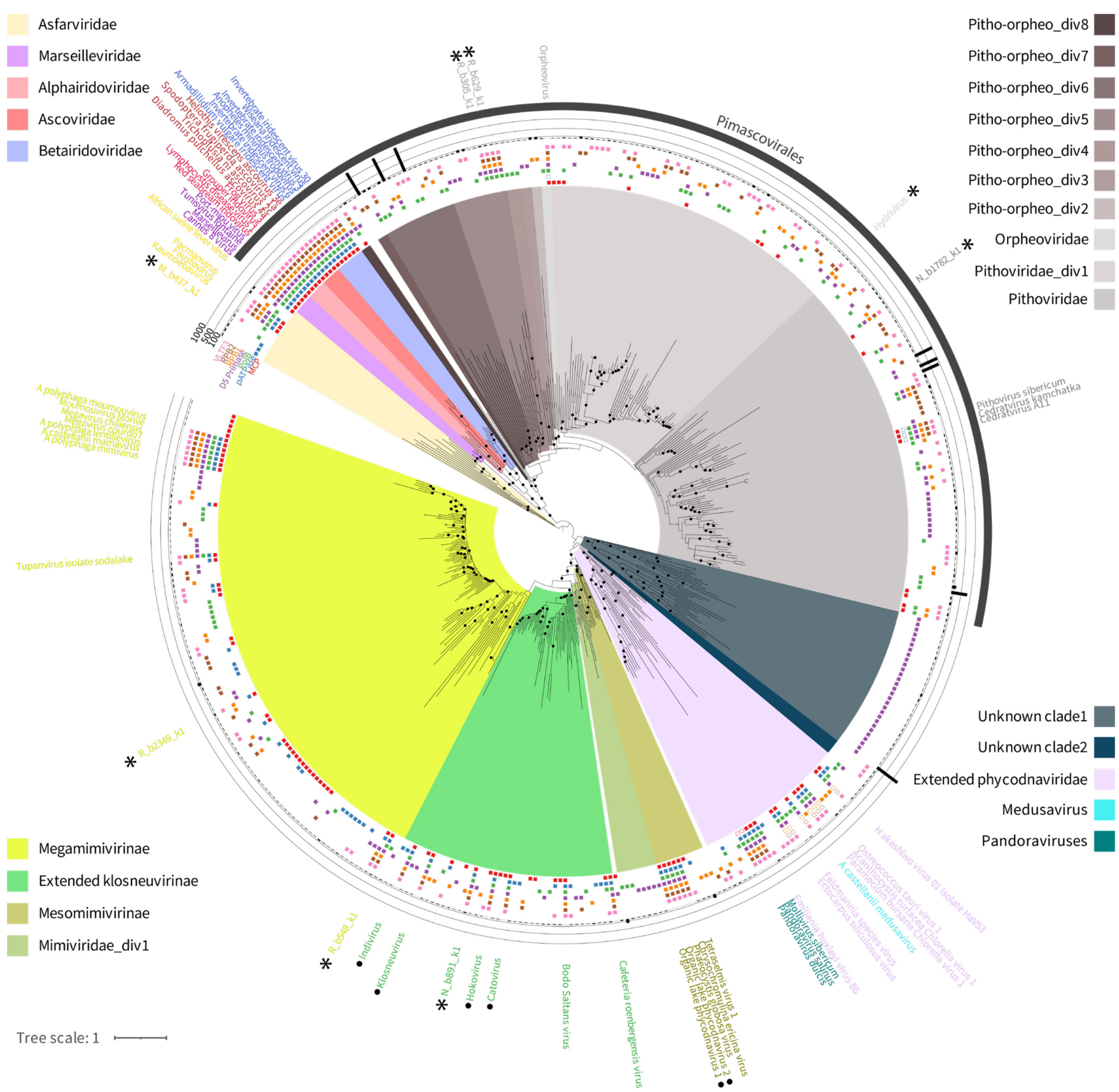

Supplementary Figure 7. ***Nucleocytoviricota*** **phylogeny in all samples**

Consensus of 1000 bootstrapped trees calculated by IQ-TREE through a partitioned analysis on 7 marker genes. The models used were the following: LG+F+R5 for the packaging ATPase and the MCP, LG+F+R6 for the RNA polymerase subunits RPB1 and RPB2, LG+F+R7 for the primase D5, LG+R+R10 for the DNA polymerase B and VT+F+R7 for VLTF3. Black dots represent branch bootstrap support ≥ 90. The colored labels indicate the reference genomes and the large genome fragments identified in this study (marked with a star). One should note that reference genomes coming from bins of previous metagenomic studies (marked with a black dot) are less reliable than the genomes of isolated viruses. The colored clades were manually created to be monophyletic. The marker genes used for this phylogeny are indicated as colored squares. Empty squares correspond to marker genes absent from the reference genomes. Black bars show the normalized mean coverage of the scaffolds. *Pimascovirales* are defined as the clade composed of all the *Ascoviridae*, *Iridoviridae*, *Marseilleviridae*, but also *Pithoviridae*, *Orpheoviridae* and the metagenomic intermediate clades. The Extended_phycodnaviridae group includes *Pandoraviridae* and Mollivirus. The Extended_klosneuvirinae group includes the Cafeteria roenbergensis virus.

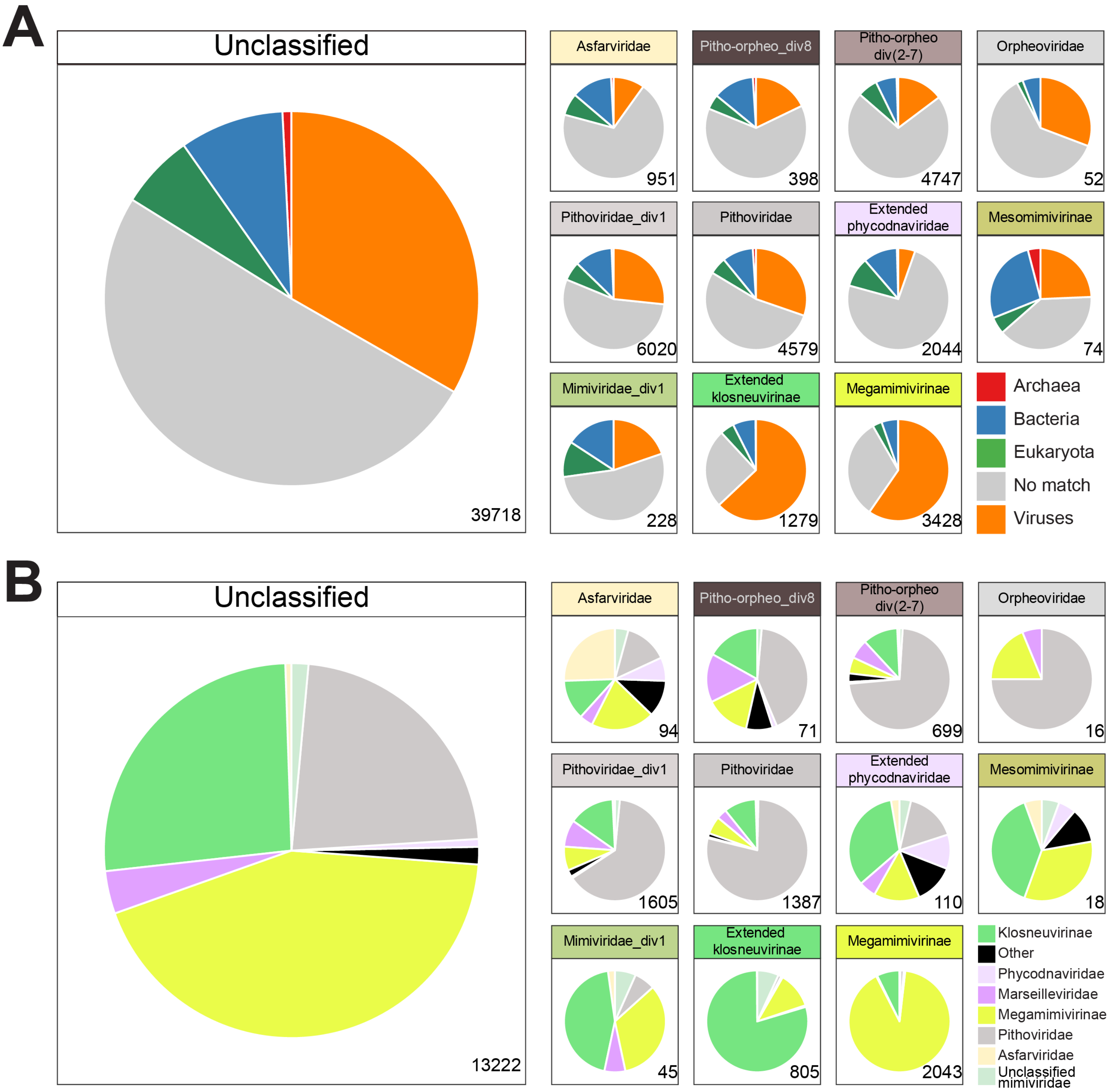

Supplementary Figure 8. Best BLASTP matches against the nr database
Pie charts of (A) best domain matches and (B) best viral matches for all permafrost sequences within phylogenetically defined groups. Total counts are shown at the bottom right corners. Source data are provided as a Source Data file.

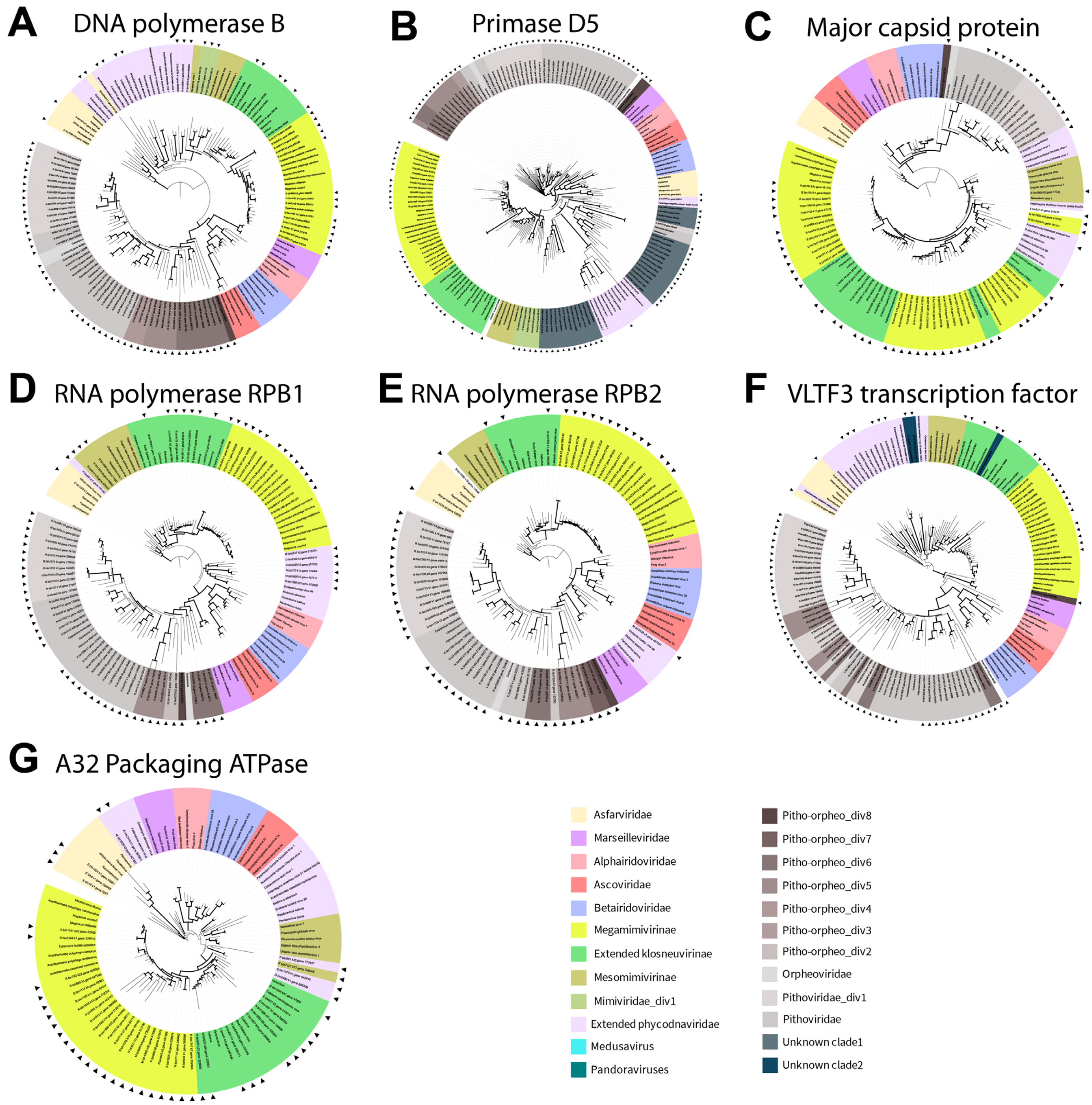

Supplementary Figure 9. Phylogenetic trees of individual *Nucleocytoviricota* marker genes

The same sequences than for the main phylogeny (Fig. 3) were used. The following models were used: LG+R+R10 for the DNA polymerase B (A), LG+F+R7 for the primase D5 (B), LG+F+R5 for the MCP (C) and the packaging ATPase (G), LG+F+R6 for the RNA polymerase subunits RPB1 (D) and RPB2 (E) and VT+F+R7 for VLTF3 (F). The wider branches indicate a support value above 80%. Permafrost sequences are marked with a black triangle.

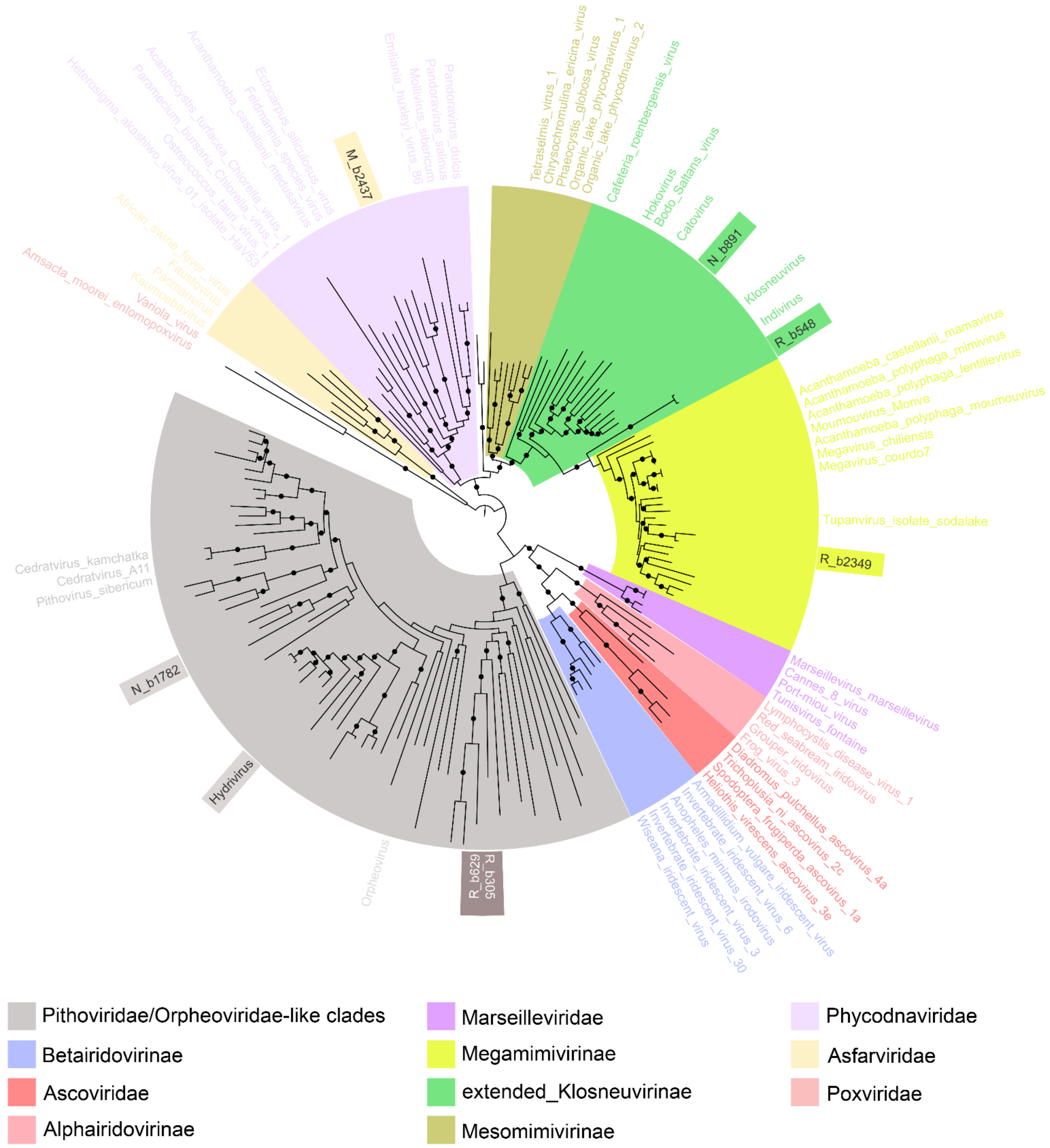

Supplementary Figure 10. Phylogenetic tree on permafrost bins

Bins containing at least 3 marker genes were used for phylogeny. Only scaffolds that have previously passed the filter as for being a *Nucleocytoviricota* have been considered. Bins containing the large scaffolds (>500 kb) identified in this study (Fig. 4) have been labeled with a box of the color of their original scaffolds' taxonomies based on Supplementary Figure 8. Reference genomes are also labeled with white background. Of notice, the M_b2437 bin assigned to *Phycodnaviridae* contains three scaffolds with different taxonomies, indicative of potential chimerism. The black circles indicate branches that are well supported (>=90% of bootstraps).

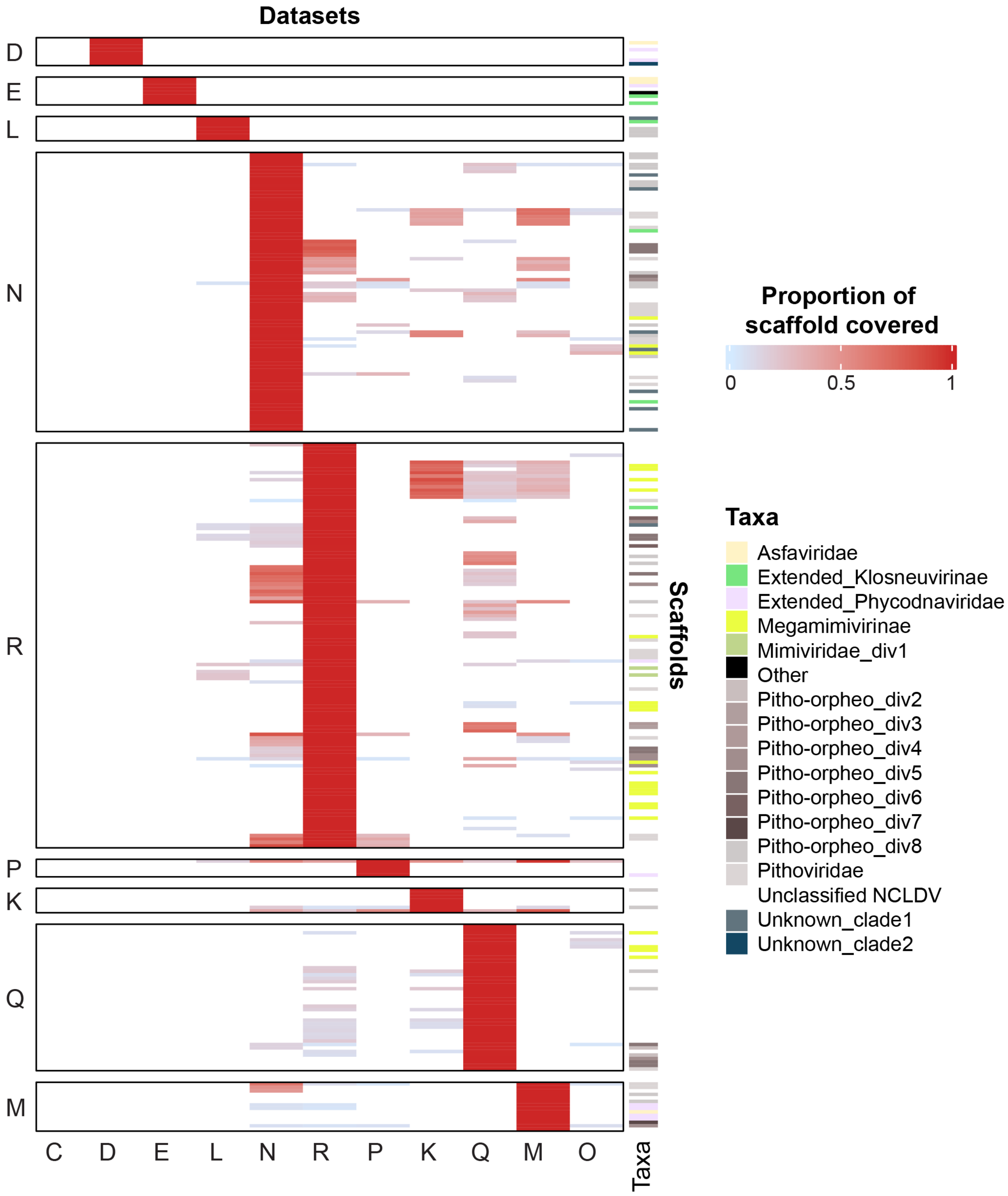

Supplementary Figure 11. Mapping of *Nucleocytoviricota* found in all samples
Reads of all samples were mapped on *Nucleocytoviricota* scaffolds over 50kb. The fraction of scaffold covered was calculated as the number of base pairs with at least one read mapped divided by the total scaffold length. White boxes indicate contigs with less than 10kb covered, considered as noise.

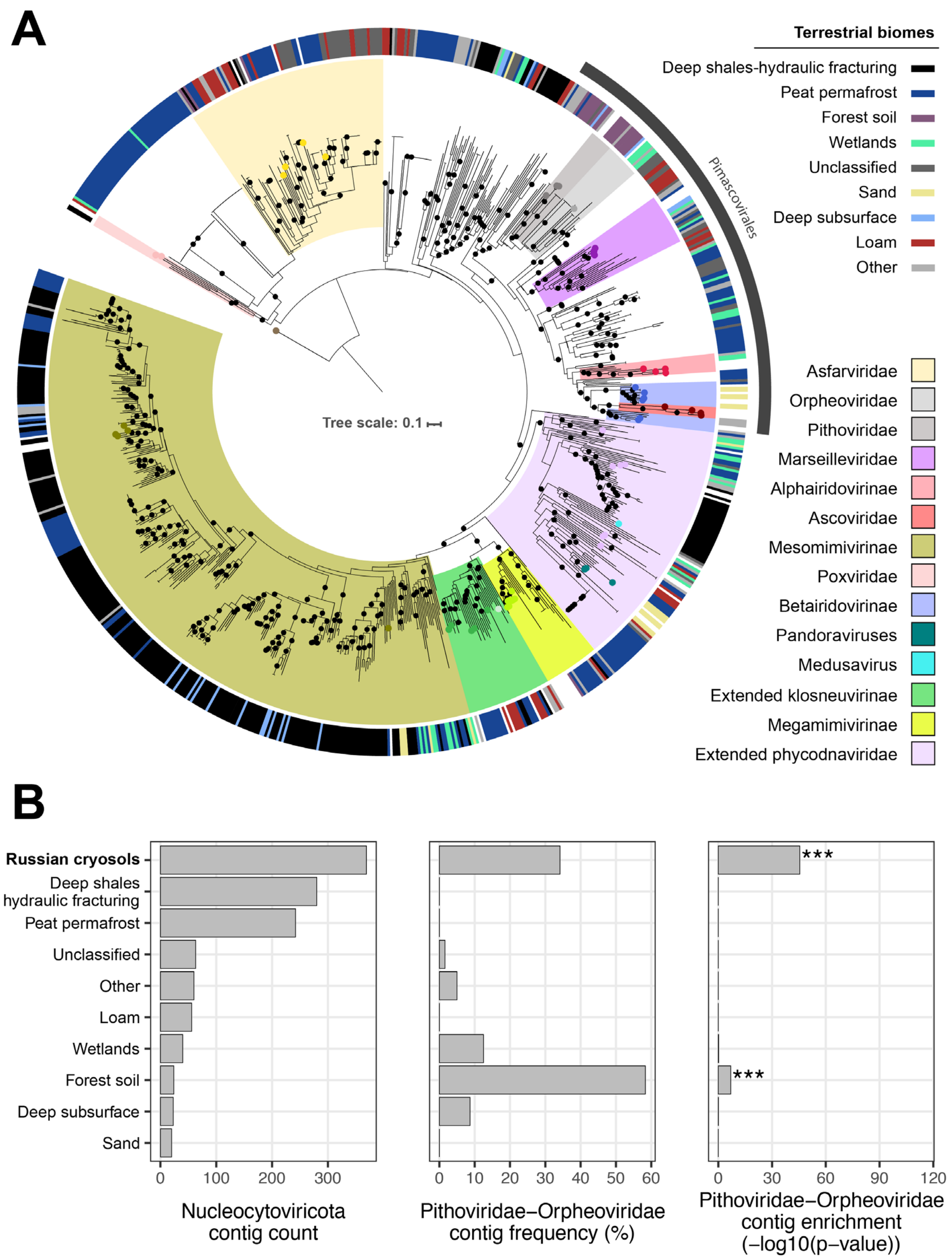

Supplementary Figure 12. *Nucleocytoviricota* recovered from terrestrial samples of the IMG/M database

(A) 804 contigs assembled from 147 terrestrial datasets of the JGI IMG/M database. Viral contigs were detected using the previously described method and placed on tree using at least one of the seven marker genes. The tree was made using Cyprinid herpesvirus 2 as outgroup. Clades containing the reference sequences were manually drawn. Colored circles at tips represent reference genomes and the outer circle shows the corresponding biome. (B) *Pithoviridae* and *Orpheoviridae* enrichment in various biomes: *Nucleocytoviricota* contig counts from our metagenomic data (Russian cryosols) and JGI samples (see Methods). The enrichment of *Pithoviridae* and *Orpheoviridae* was calculated through an asymmetrical Fisher test per biome, counting *Orpheoviridae* and *Pithoviridae* versus other *Nucleocytoviricota* among the tested biome and all other biomes.

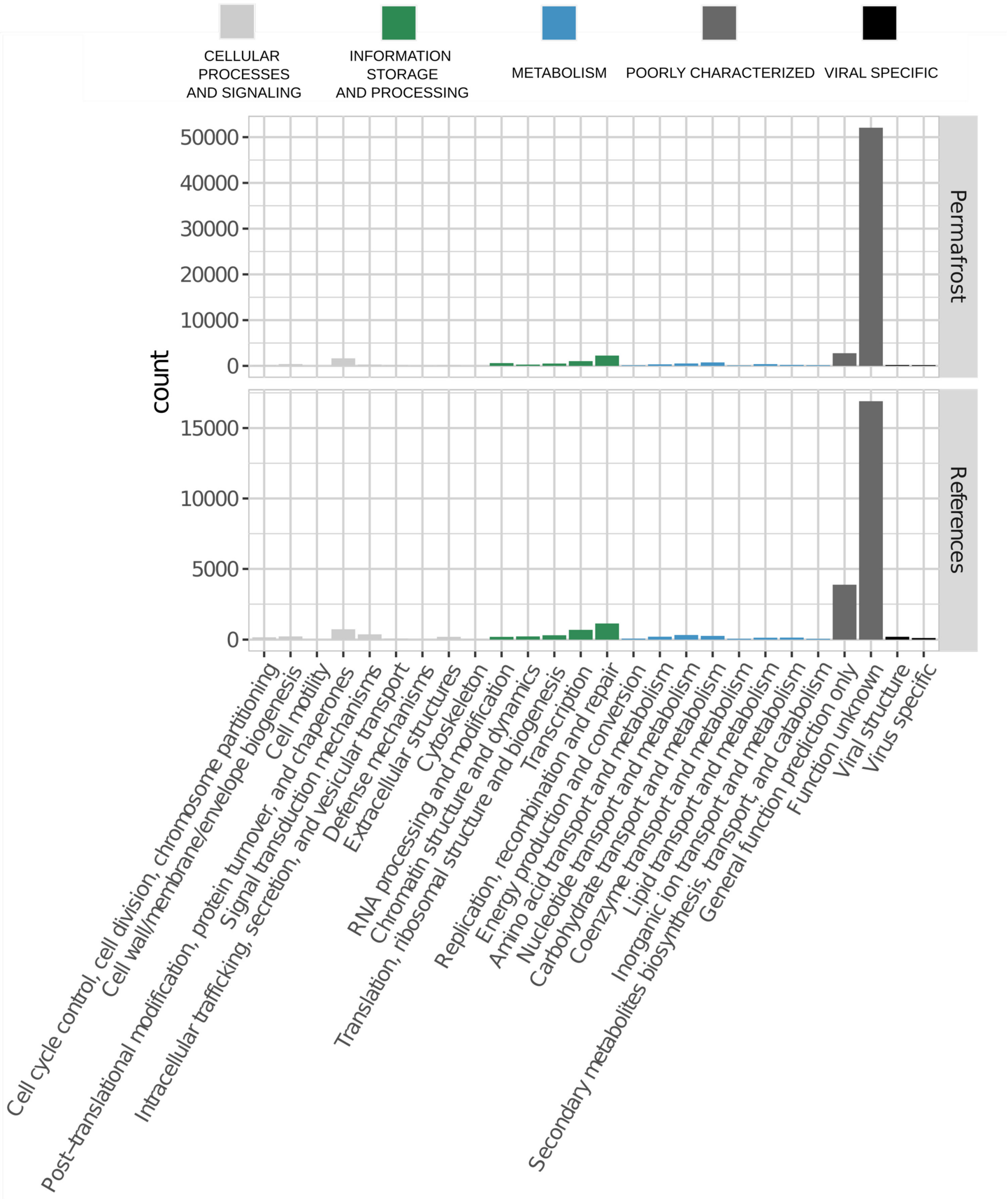

Supplementary Figure 13. Functional categories of predicted *Nucleocytoviricota* ORFs
Viral ORFs from the Russian cryosol metagenomes were manually functionally annotated and assigned to a category. Source data are provided as a Source Data file.

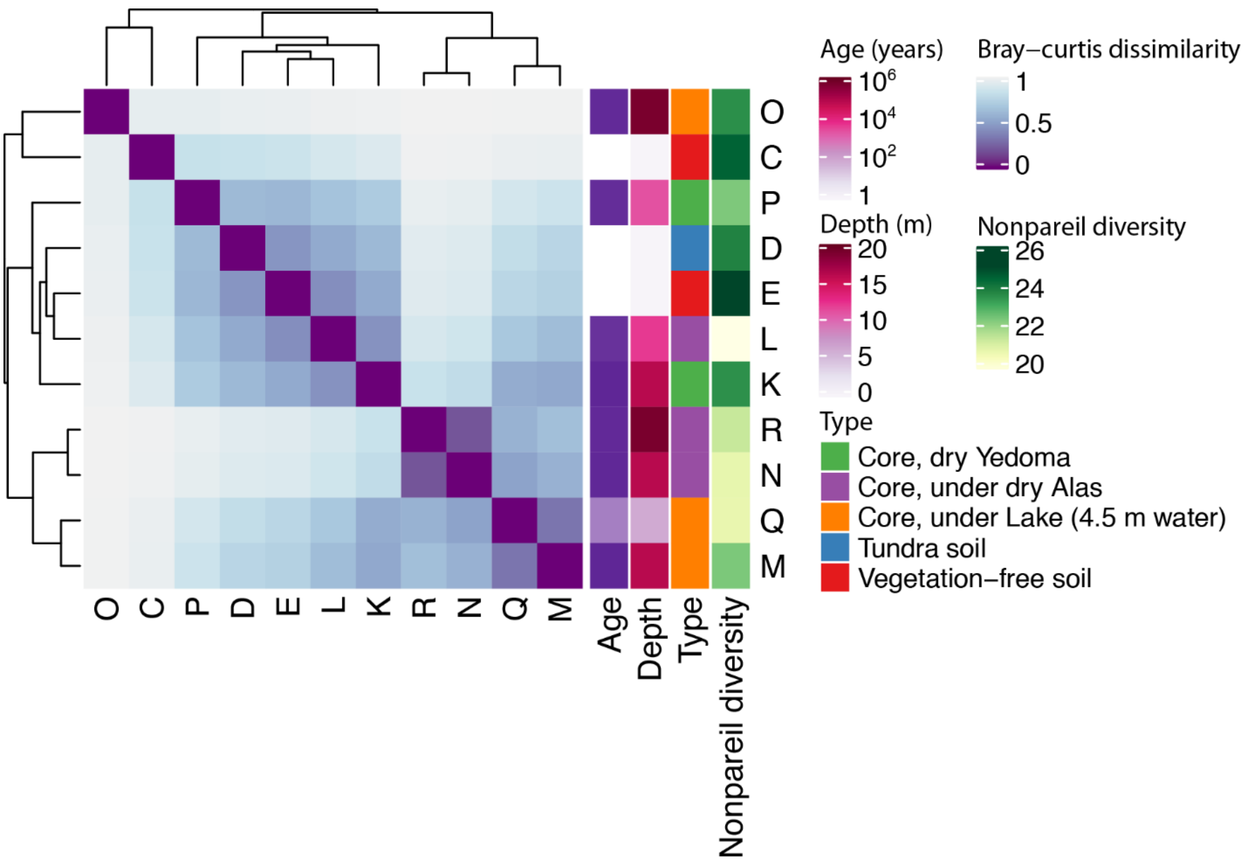

Supplementary Figure 14. Samples clustered according to the viral functional content. The heatmap presents samples clustered based in the Bray-Curtis dissimilarity calculated on Pfam annotations occurrences. Some ecological parameters are shown for each sample.

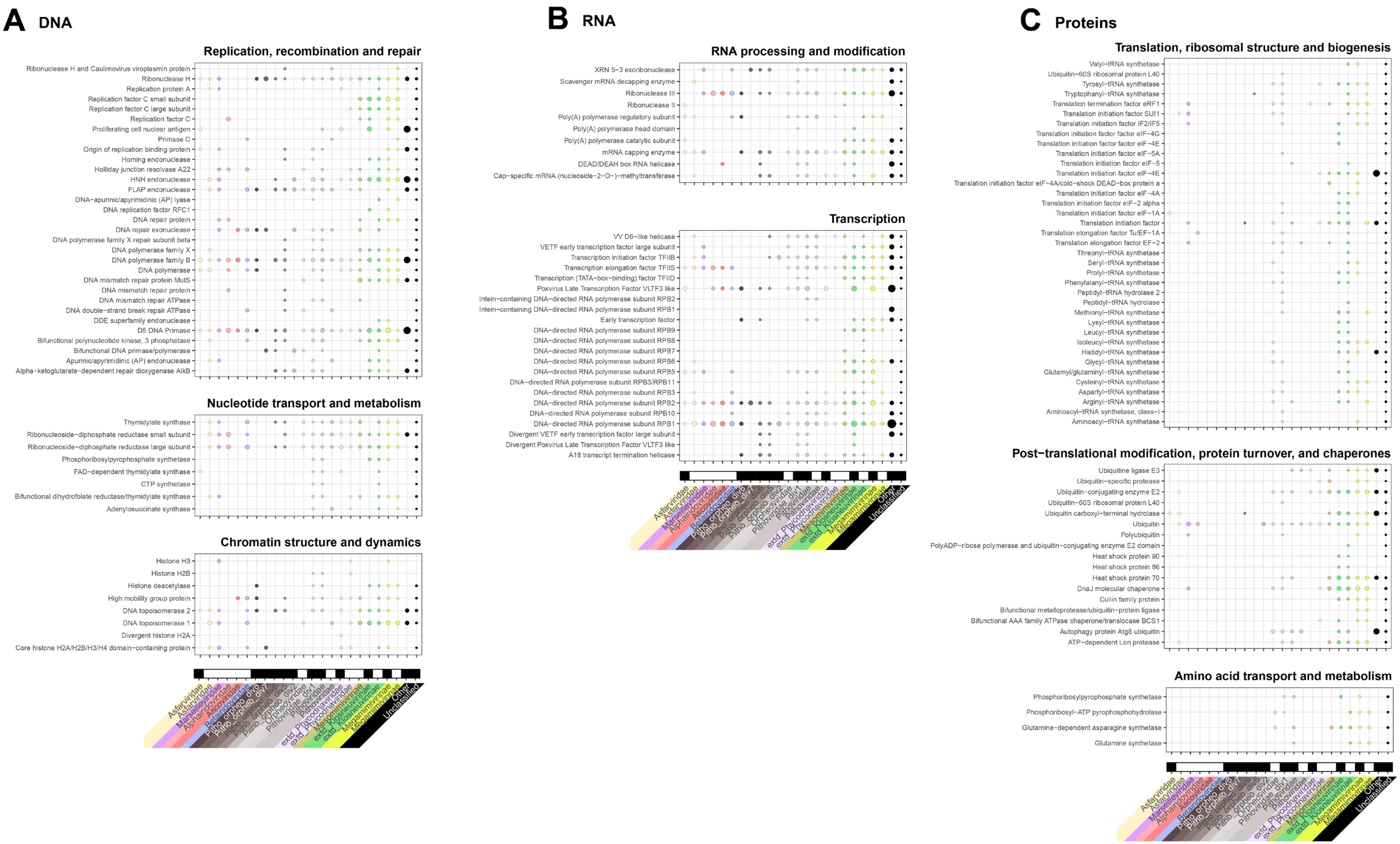

Supplementary Figure 15. Relative copy number of ORFs of selected functions within reference and permafrost *Nucleocytoviricota*

The bubbles sizes represent the relative number of copies of the functions found within a particular family. The bottom squares represent reference (white) or metagenomic (black) groups. The category "Other" includes families with less than 250 ORFs. Functions were separated according to the ones concerning DNA (A), RNA (B) or proteins (C).

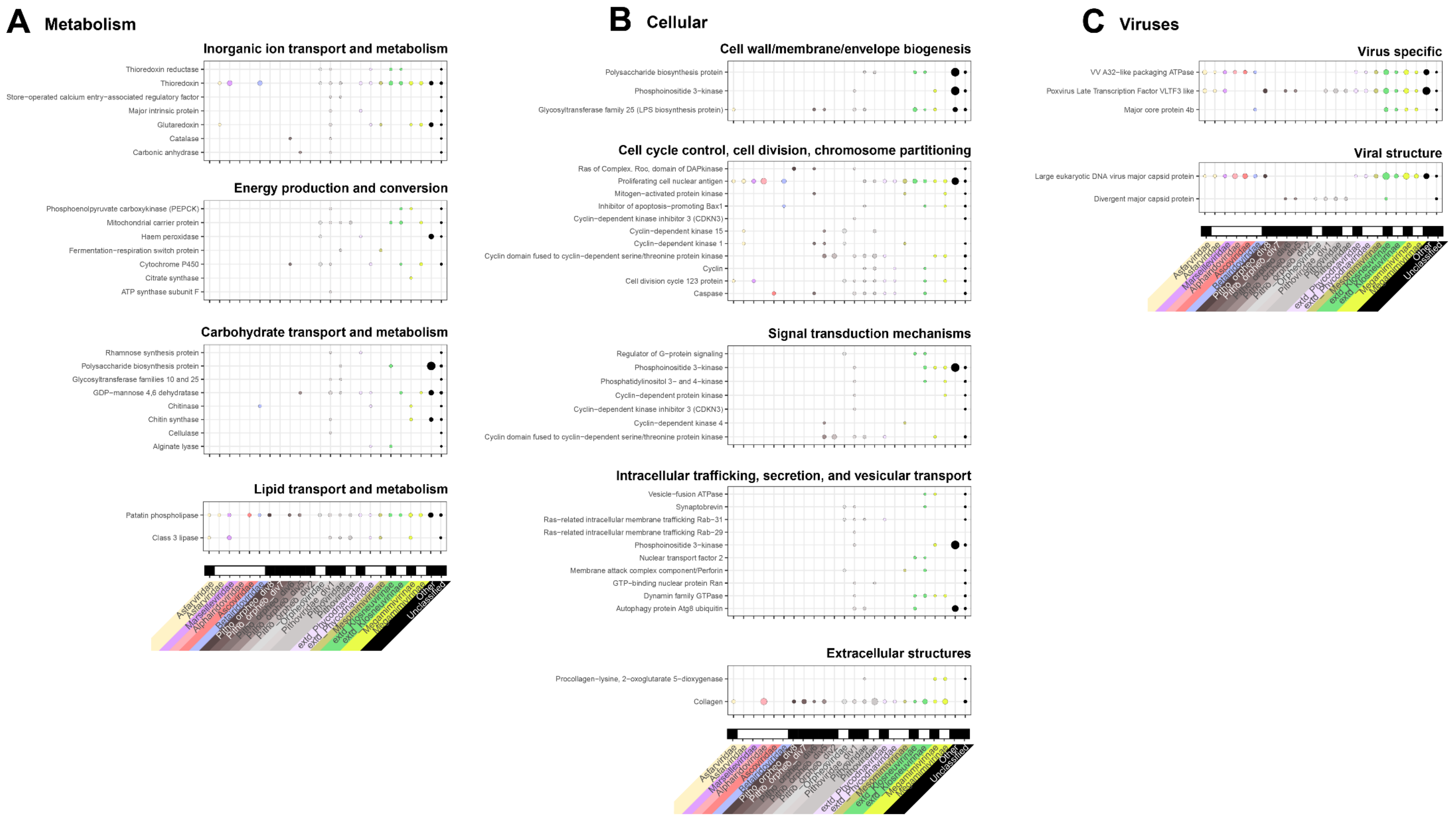

Supplementary Figure 16. Relative copy number of ORFs of selected functions within reference and permafrost *Nucleocytoviricota*

The bubbles sizes represent the relative number of copies of the functions found within a particular family. The bottom squares represent reference (white) or metagenomic (black) groups. The category "Other" includes families with less than 250 ORFs. Functions were separated into metabolism (A), cellular functions (B) and viral functions (C).

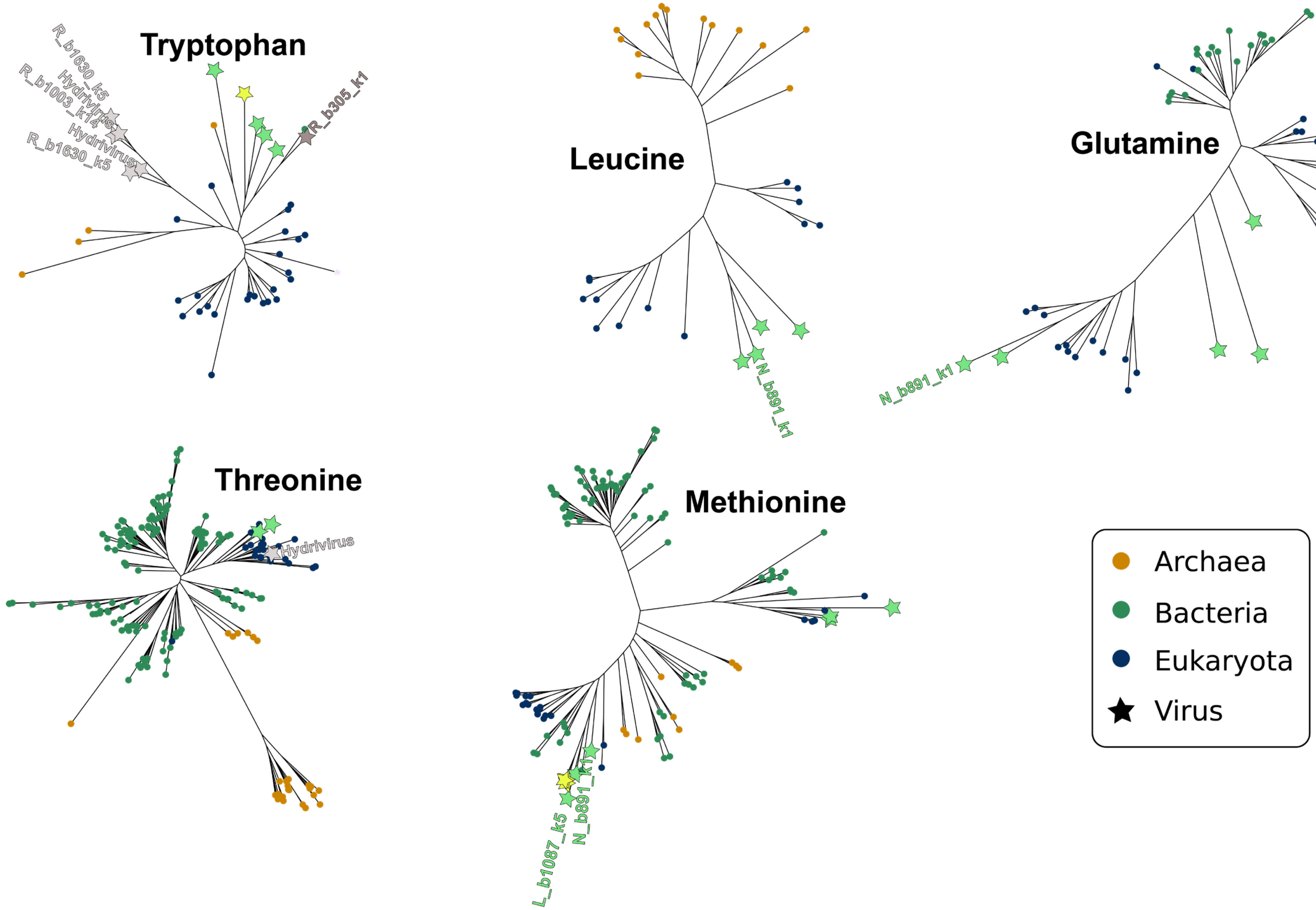

Supplementary Figure 17. Evidence of Aminoacyl-tRNA synthases exchanged between Eukaryota and *Nucleocytoviricota*
All trees are monophyletic according to their larger phylogenies (see Methods). Aminoacyl-tRNA synthetases were retrieved from IPR001412 and IPR006195 InterPro families to which were added *Nucleocytoviricota* sequence and their respective matches against the nr database.

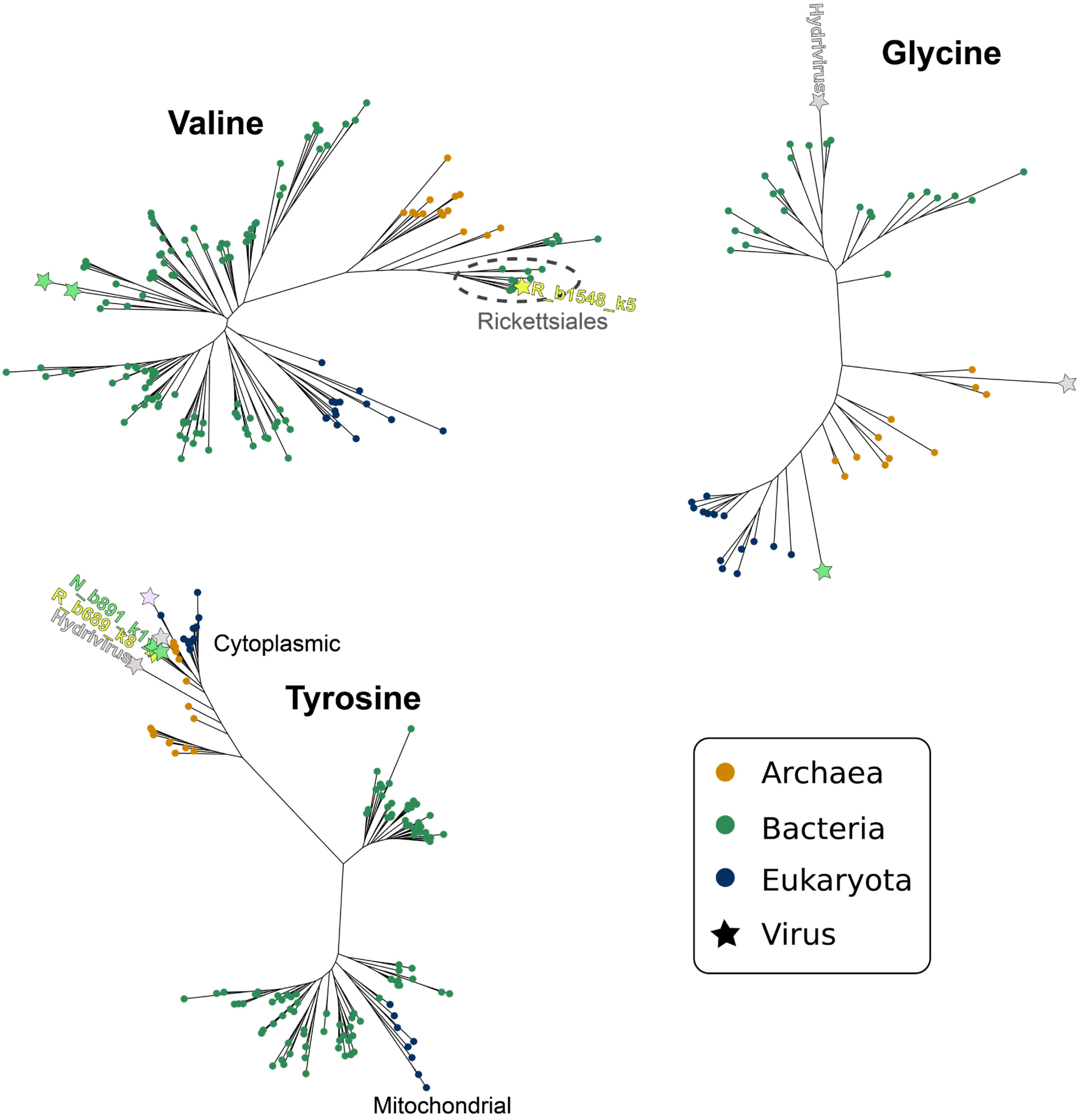

Supplementary Figure 18. Evidence of Aminoacyl-tRNA synthases exchanged between Prokaryotes and *Nucleocytoviricota*

All trees are monophyletic according to their larger phylogenies (see Methods). Aminoacyl-tRNA synthetases were retrieved from IPR001412 and IPR006195 InterPro families to which were added *Nucleocytoviricota* sequence and their respective matches against the nr database.

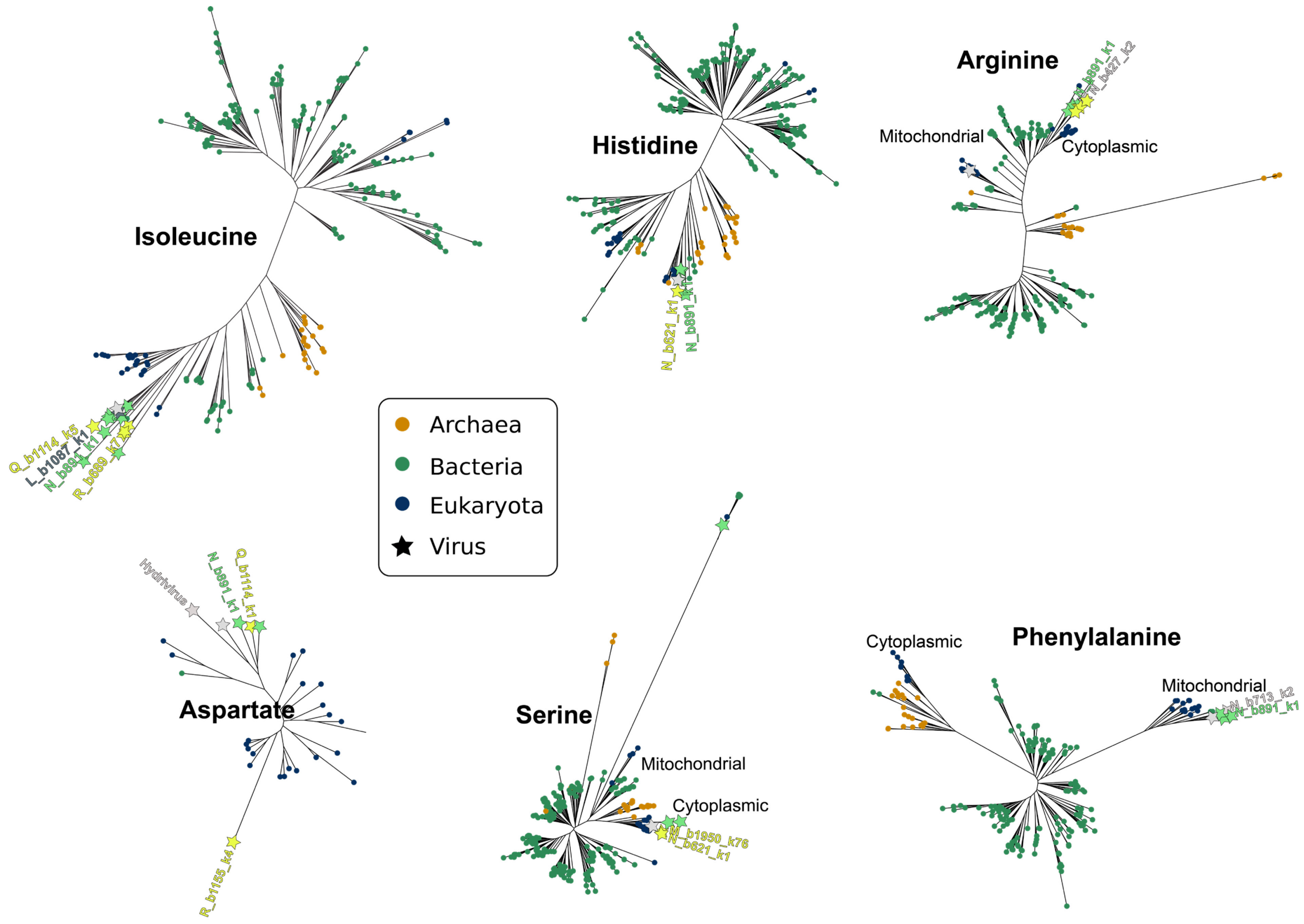

Supplementary Figure 19. Potential Aminoacyl-tRNA synthase exchanges between viruses
All trees are monophyletic according to their larger phylogenies (see Methods). Aminoacyl-tRNA synthetases were retrieved from IPR001412 and IPR006195 InterPro families to which were added *Nucleocytoviricota* sequence and their respective matches against the nr database.

Supplementary Table 1. Analyzed permafrost datasets

Cryosol type	Sample code	Locality	Type	Depth (m)	Dating (103 y)	Million reads	Dataset accession
Kamchatka (surface)	C	Kronotsky river bank	Vegetation-free soil	0	0	677	ERS7649018
	D	Kizimen volcano	Tundra soil	0	0	642	ERS7649019
	E	Shapina river bank	Vegetation-free soil	0	0	685	ERS7649020
Permafrost (Yukechi Alas area)	L	Under dry Alas	Core	12	28	645	ERS7649022
	N			16	45	675	ERS7649024
	R			19	42	628	ERS7649028
	P	Dry Yedoma		11	36	616	ERS7649026
	K			16	49	569	ERS7649021
	Q	Under lake (4.5m water)		6	0.053	635	ERS7649027
	M			16	48.5	649	ERS7649023
	O			19	40	586	ERS7649025

Supplementary Table 2. Assembly statistics all datasets combined

The statistical test used to compare both assemblies in contig length is a two-sided Wilcoxon rank sum test.

	Contigs over 1 kb			Contigs over 10 kb		
	**Total assembled nt**	**Contigs**	**Mean (median) length**	**Total assembled nt**	**Contigs**	**Mean (median) length**
**1st assembly round**	1.97E-10	8,698,362	2,265 (1,473)	3.14E9	149,459	20,983 (14,660)
**2nd assembly round**	1.21E-10	2,720,241	4,432 (2,448)	4.51E9	185,996	24,274 (15,607)
**P-value Wilcoxon test (2nd vs 1st assemblies)**			<2.2E-16			<2.2E-16

Supplementary Table 3. Evalue thresholds calculated to select marker genes for phylogeny through psiblast

Marker gene	Evalue threshold
mcp	2.3875E-45
pATPase	1.72E-31
polB	1.67E-53
primase	1.4675E-36
rpo1	1.36E-68
rpo2	1.79E-74
vltF3	0.000000001065
