# Supplementary material for: Past and present giant viruses diversity explored through permafrost metagenomics
Source: Nat Commun. 2022 Oct 7;13:5853. doi: 10.1038/s41467-022-33633-x (PMC9546926; doi:10.1038/s41467-022-33633-x)
Supplement: Supplementary file 3 — Description of Additional Supplementary Files [file 41467_2022_33633_MOESM3_ESM.pdf]

File Name: Supplementary Data 1
Description: Nucleocytoviricota core genes used in this study

File Name: Supplementary Data 2
Description: Pfam annotations significantly enriched in the permafrost viruses compared to reference Nucleocytoviricota

File Name: Supplementary Data 3
Description: Annotations significantly enriched in given samples compared to the others

File Name: Supplementary Data 4
Description: Control dataset composition

File Name: Supplementary Data 5
Description: Fasta files of permafrost *Nucleocytoviricota* sequences and additional HMM files

File Name: Supplementary Data 6
Description: JGI IMG/M terrestrial metagenomes analyzed
