# Supplementary material for: Past and present giant viruses diversity explored through permafrost metagenomics
Source: Nat Commun. 2022 Oct 7;13:5853. doi: 10.1038/s41467-022-33633-x (PMC9546926; doi:10.1038/s41467-022-33633-x)
Supplement: Supplementary file 10 — Reporting Summary [file 41467_2022_33633_MOESM10_ESM.pdf]

nature portfolio

Corresponding author(s): Matthieu LEGENDRE

Last updated by author(s): Sep 15, 2022

Reporting Summary

Nature Portfolio wishes to improve the reproducibility of the work that we publish. This form provides structure for consistency and transparency in reporting. For further information on Nature Portfolio policies, see our Editorial Policies and the Editorial Policy Checklist.

Statistics

For all statistical analyses, confirm that the following items are present in the figure legend, table legend, main text, or Methods section.

n/a	Confirmed	
☐	☒	The exact sample size (*n*) for each experimental group/condition, given as a discrete number and unit of measurement
☐	☒	A statement on whether measurements were taken from distinct samples or whether the same sample was measured repeatedly
☐	☒	The statistical test(s) used AND whether they are one- or two-sided<br>*Only common tests should be described solely by name; describe more complex techniques in the Methods section.*
☒	☐	A description of all covariates tested
☐	☒	A description of any assumptions or corrections, such as tests of normality and adjustment for multiple comparisons
☐	☒	A full description of the statistical parameters including central tendency (e.g. means) or other basic estimates (e.g. regression coefficient) AND variation (e.g. standard deviation) or associated estimates of uncertainty (e.g. confidence intervals)
☐	☒	For null hypothesis testing, the test statistic (e.g. *F*, *t*, *r*) with confidence intervals, effect sizes, degrees of freedom and *P* value noted<br>*Give P values as exact values whenever suitable.*
☒	☐	For Bayesian analysis, information on the choice of priors and Markov chain Monte Carlo settings
☒	☐	For hierarchical and complex designs, identification of the appropriate level for tests and full reporting of outcomes
☒	☐	Estimates of effect sizes (e.g. Cohen's *d*, Pearson's *r*), indicating how they were calculated

Our web collection on statistics for biologists contains articles on many of the points above.

Software and code

Policy information about availability of computer code

Data collection	
Data analysis	Softwares used in the study:<br>Spades (v3.14)<br>Metabat2 (v2.15)<br>Bowtie2 (v2.3.4.1)<br>Checkm (v1.1.2)<br>Genemark (v3.36)<br>BLAST+ (v2.8.1)<br>Hmmer suite (v3.2.1)<br>TopGO (v2.40.0)<br>Diamond (v0.9.31.132)<br>Interproscan (v.5.39-77)<br>Emapper-1.03<br>Barrnap (v0.9)<br>Viralrecall (v2.0)<br>Integrative Genome Viewer (2.8.6)<br>Samtools (v1.3.1)<br>MAFFT (v7.407)<br>ClipKIT (v1.3.0)

Iqtree (v1.6.12)
PASTA (v1.9.0)
Catsequences (v1.3)
Itol (v6.5.1)
CDhit (v4.8.1)
Modelestimator (v1)
"Custom scripts codes used in this study can be accessed here: https://doi.org/10.6084/m9.figshare.20101850"

For manuscripts utilizing custom algorithms or software that are central to the research but not yet described in published literature, software must be made available to editors and reviewers. We strongly encourage code deposition in a community repository (e.g. GitHub). See the Nature Portfolio guidelines for submitting code & software for further information.

Data

Policy information about availability of data

All manuscripts must include a data availability statement. This statement should provide the following information, where applicable:

- Accession codes, unique identifiers, or web links for publicly available datasets
- A description of any restrictions on data availability
- For clinical datasets or third party data, please ensure that the statement adheres to our policy

Supplementary data files can be accessed here: https://doi.org/10.6084/m9.figshare.19464917. Large genome fragments and annotations were deposited under the study PRJEB47746 with the following accessions: ERS10539964, ERS10539963, ERS10539962, ERS10539961, ERS10539960, ERS10539959, ERS10539958, ERS10539957. Source data are provided with this paper. In addition, previously published public data used for analysis includes: Genbank NR (from June 2020), GVMAGs (https://figshare.com/s/14788165283d65466732) and https://genome.jgi.doe.gov/portal/GVMAGs/GVMAGs.home.html), VOG orthogroups (https://vogdb.org/), Refseq protein database (from March 2020), EggNOG (v5), GEVEs (https://zenodo.org/record/3975964#.XzFj0hl7mfZ), JGI IMG/M (database 2021, https://img.jgi.doe.gov/).

Field-specific reporting

Please select the one below that is the best fit for your research. If you are not sure, read the appropriate sections before making your selection.

☐ Life sciences ☐ Behavioural & social sciences ☒ Ecological, evolutionary & environmental sciences

For a reference copy of the document with all sections, see nature.com/documents/nr-reporting-summary-flat.pdf

Ecological, evolutionary & environmental sciences study design

All studies must disclose on these points even when the disclosure is negative.

Study description	The study provides computational analyses of large DNA viral genomes in permafrost metagenomics data and comparison to other environnments. No replicates were used in this study.
Research sample	Among the 12 permafrost samples generated by a previous study (https://doi.org/10.1093/femsml/uqac003), eleven were kept for analysis. One sample (sample B) was excluded since it was older than the other ones and contained less data with shorter reads. For comparison, other terrestrial samples were also collected from the JGI IMG/M databases. All available samples with contigs over 10 kb were analyzed.
Sampling strategy	As described in (https://doi.org/10.1093/femsml/uqac003) no statistical method was used for the sampling procedure. We used samples corresponding to 3 different forages and two to three samples by forage. We were able to reveal a large variety of Nucleocytoviricota sequences even with this small dataset.
Data collection	Data used in this study were retrieved from a previous work (https://doi.org/10.1093/femsml/uqac003). These samples were collected by Alexander Morawitz. Furthermore Dr. Jens Strauss and Dr. Guido Grosse performed the forage, storage and transportation of the samples.
Timing and spatial scale	As described in the previous study (https://doi.org/10.1093/femsml/uqac003) for which samples were collected, the sampling took place in July 2017 and March 15, as part of a programmed expedition considering climatic and administrative constraints.<br>The surface cryosol samples were collected on July 2017 at these locations (GPS coordinates):<br>- sample C: 54.549722°N, 160.581944°E<br>- sample D: 55.098611°N, 160.349444°E<br>- sample E: 55.115°N, 159.963333°E<br>The permafrost samples were collected on March 2015 at these locations (GPS coordinates):<br>- samples Q, O and M: 61.76086°N, 130.47466°E<br>- samples P and K: 61.75967°N, 130.47438°E<br>- samples L, N and R: 61.76490°N, 130.46503°E
Data exclusions	One sample was excluded from the original permafrost dataset because it was sequenced using a different technology with shorter reads.
Reproducibility	The sequencing data from the previous study (https://doi.org/10.1093/femsml/uqac003) was not replicated to optimize the quantity and diversity of sequenced DNA. The present study does not contain experimental results but computational procedures to reveal

	Nucleocytoviricota sequences were applied on different datasets. Bioinformatics procedures are described in the methods section and can be replicated.
Randomization	We did not employ any randomization in this study since we did not have different treatment groups.
Blinding	Blinding was not relevant for this study as all datasets were treated the same way.

Did the study involve field work? ☐ Yes ☒ No

Reporting for specific materials, systems and methods

We require information from authors about some types of materials, experimental systems and methods used in many studies. Here, indicate whether each material, system or method listed is relevant to your study. If you are not sure if a list item applies to your research, read the appropriate section before selecting a response.

Materials & experimental systems

n/a	Involved in the study	
☒	☐	Antibodies
☒	☐	Eukaryotic cell lines
☒	☐	Palaeontology and archaeology
☒	☐	Animals and other organisms
☒	☐	Human research participants
☒	☐	Clinical data
☒	☐	Dual use research of concern

Methods

n/a	Involved in the study	
☒	☐	ChIP-seq
☒	☐	Flow cytometry
☒	☐	MRI-based neuroimaging
